# Joint Green Marketing Decision-Making of Green Supply Chain Considering Power Structure and Corporate Social Responsibility

**DOI:** 10.3390/e23050564

**Published:** 2021-05-01

**Authors:** Jialiang Huang, Xiaoxia Wang, Yuxi Luo, Liying Yu, Ziyuan Zhang

**Affiliations:** School of Management, Shanghai University, Shanghai 200444, China; dimples_hjl@126.com (J.H.); 15189837918@163.com (X.W.); luoyuxi0105@163.com (Y.L.); yuliying@shu.edu.cn (L.Y.)

**Keywords:** green supply chain, power structure, corporate social responsibility, joint green marketing, pricing decision

## Abstract

In order to explore the impact of a manufacturer’s or retailer’s undertaking corporate social responsibility (CSR) and different power structures on their joint green marketing decisions and profits in the green supply chain, this paper establishes green supply chain optimization models under six different decision-making scenarios according to two different CSR bearers and three different power structures. Based on the main assumptions of a linear product demand function and CSR measured by consumer surplus, this paper solves the equilibrium solutions of the manufacturer and the retailer through game theory. The results show that: First, the difference in the degree of CSR undertaken by manufacturers and retailers leads to a difference in the ranking of optimal strategies of both parties under the three power structures. Second, under the same power structure, compared with undertaking CSR by oneself, when the other party undertakes CSR, the level of the product’s green degree, the level of green promotion, the party’s own profit, and the profit of the other party are all higher. Third, regardless of the power structure, manufacturers and retailers undertaking CSR is conducive to improving the level of product greenness, increasing green promotion, lowering the retail price, increasing consumers’ willingness to buy green products, and ultimately helping to increase the profits of manufacturers and retailers.

## 1. Introduction

With rapid economic development, the increasing scarcity of natural resources and the further aggravation of environmental pollution have accelerated the transformation of economic development mode transformation. Achieving green and sustainable development has become a hot topic of concern for governments, enterprises, and scholars. The advent of the green era poses new challenges to the management of enterprise production and operation. The green supply chain management model, which fully considers resource consumption and environmental impact based on the supply chain, has received wide attention from all walks of life. Simultaneously, with the vigorous promotion of ecological civilization construction and the continuous improvement of consumers’ environmental awareness, green products are gradually becoming favored by the market, and their market demand is gradually expanding. Implementing green supply chain management and actively producing and selling green products have become important measures for supply chain enterprises to occupy a favorable market position and obtain sustainable competitive advantages, so it is of great practical significance to explore green supply chain member enterprises’ green marketing decisions at this stage.

As consumers’ perception of green consumption grows stronger, more and more manufactures in the supply chain are working together with retailers in order to attract potential consumers. On the one hand, manufactures have taken steps to produce products with higher green degrees. For example, Best Buy, the world’s largest home appliance retailer, saves energy and protects the environment by selling Energy Star, a product with superior energy efficiency [1]. On the other hand, retailers implement green promotion to attract consumers to purchase green products. For example, Walmart sets up special areas in shopping malls to develop green lifestyle education promotion activities by cooperating with suppliers [1]. The above examples and numerous studies have confirmed that cooperation between manufacturers and retailers in green marketing activities has become an effective way of marketing. Therefore, it is of great practical significance to study the supply chain members’ joint green marketing issues in the context of the green economy.

In the context of sustainable development, corporate social responsibility (CSR) is closely linked to sustainable development. To achieve comprehensive sustainable development, supply chain members need to take corresponding CSR while pursuing economic benefits. CSR refers to enterprises’ responsibility to the environment and their stakeholders; therefore, in addition to considering their own profits and operating conditions, enterprises must also consider the impact of their operations on the natural environment and society. Generally speaking, corporate social responsibility mainly includes environmental responsibility and social (welfare) responsibility. In the green supply chain, since manufacturers and retailers jointly carrying out green marketing activities have already taken corresponding environmental responsibilities, the corporate social responsibility referred to by green supply chain companies refers to the behavior of companies paying attention to stakeholders such as consumers. For example, Tesco, as a large retailer, has established a production quality management system to ensure food safety and improved various systems to protect consumers’ rights [2]. With the enhancement of consumers’ social consciousness, more and more consumers are more inclined to choose companies that actively undertake corporate social responsibilities, and CSR characteristics are becoming a critical antecedent factor for companies to gain consumer recognition. However, an enterprise is an organization whose goal is to maximize profit; when it undertakes corporate social responsibility in the supply chain, it comprehensively considers the cost of responsibility and corporate profits. Therefore, the corporate social responsibility of supply chain members affects the game among members and further affects their decision-making. 

In real life, there are different channel powers among members of the green supply chain. The more powerful party often occupies a dominant position in the supply chain, and it has a more significant advantage in the decision-making process and can dominate the direction of the supply chain. Generally speaking, there are three different power structures in the supply chain: manufacturer-led, retailer-led, and co-led by both parties. The channel power structure also affects the game among supply chain members and further affects the corresponding decision-making. Therefore, in the case of complex and diverse power structures, it is of great practical significance to explore the impact of corporate social responsibility on the joint green marketing strategies of green supply chain enterprises.

Based on the above background, this paper takes the green supply chain as the research object, and studies the joint green marketing decision problem of the manufacturer and retailer considering different power structures and both parties’ awareness of corporate social responsibility by using the Stackelberg game method. We were mainly committed to solving the following problems:

(1) How do different power structures affect the optimal pricing strategy, green marketing strategy, and profits of both parties when a manufacturer or retailer takes on corporate social responsibility?
(2) What is the impact of different power structures on the two parties’ optimal pricing strategies, green marketing strategies, and profits with the same corporate social responsibility bearer?
(3) What is the impact of different corporate social responsibility bearers on the two parties’ optimal pricing strategies, green marketing strategies, and profits with the same power structure?

The main contributions of this paper are as follows:

First, this paper integrated CSR and power structures to discuss manufacturers’ and retailers’ joint green marketing decisions. Second, as upstream manufacturers and downstream retailers start from undertaking CSR and carrying out joint green marketing, from each other’s leading to self-leading, and then to co-leading, the decision points of both parties begin to change from indefinite to restrictive. This is the process of entropy reduction. Finally, this paper adopted game theory, which was used as a system analysis method, an approach used to explore the process of interaction of interests between different objects in a system based on specific rules. This interaction process must be accompanied by an increase in entropy of one party and a decrease in entropy of the other party. In conclusion, this paper thoroughly and comprehensively explores the impact of different members’ CSR awareness and power structures from the perspective of entropy, which can provide theoretical support for cooperative green marketing of green supply chain members from relevant perspectives.

This paper is organized as follows. Section 2 is the literature review. Section 3 provides problem description and assumptions. The optimal decisions under six decision scenarios are presented in Section 4. Section 5 is comparison and analysis section, which summarizes some research conclusions by comparing the equilibrium solutions obtained from Section 4. Section 6 gives some numerical examples to test and verify the findings. Conclusions and limitations of the study are summed up in Section 7.

## 2. Literature Review

This section mainly sorts out three types of literature related to supply chain operation decision issues in terms of research content. The first type is about operation decision issues for the green supply chain. The second type is about supply chain operation decision issues considering CSR. The third type is about supply chain operation decision issues considering power structure.

At present, the operational decision-making issues of the green supply chain have been deeply studied [3,4]. In terms of pricing decisions, Zhang et al. [5] explored green supply chain optimal pricing decisions considering fairness concerns under government subsidies. Chen et al. [6] and Zhu and He [7] studied the pricing problem of green products in the presence of competing manufacturers and retailers, respectively. Sang [8] and Yang and Xiao [9] investigated the problem of optimal pricing strategies for green products in green supply chains under fuzzy market demand. Li et al. [10] studied the impact of product pricing decisions in a secondary green supply chain consisting of a fair–neutral manufacturer and a retailer with fairness concerns. In terms of green supply chain coordination studies, Swami and Shah [11] coordinated two levels of green supply chains by means of two pricing contracts, while Hong and Guo [12] used burden-sharing contracts and Song and Gao [13] used revenue-sharing contracts to coordinate the green supply chain system and pointed out that the profits of both manufacturers and retailers could be improved under these two contracts, respectively.

With the increasing social awareness of consumers, enterprises have to actively develop and implement CSR for their supply chain systems to win sustainable competitive advantages [14]. Moreover, the production and operation of enterprises cannot only aim at their own profit but also need to take the responsibility of contributing to the environment, consumers, and society. There are currently scholars in various areas who are introducing CSR into supply chain management and decision-making issues. In terms of supply chain coordination, Ni and Li [15] considered that CSR, in sequential and simultaneous decision-making by supply chain node firms to undertake CSR scenarios, could lead to a win–win situation for node firms with a mutual incentive mechanism. Panda [16] established a two-level supply chain consisting of a manufacturer and a retailer and found that supply chain coordination can be achieved by considering CSR only by suppliers, quantity discount contracts, revenue sharing contracts, and wholesale price discount contracts. Modak et al. [17] analyzed the pricing strategies of a manufacturer with CSR and two competing retailers and used a two-part pricing contract to coordinate the supply chain system. Then, Panda and Modak [18] used consumer surplus as a CSR measure to allow members of the supply chain to bear a portion of CSR to achieve channel coordination. Raza [19] discussed the use of revenue sharing contracts to coordinate the supply chain and achieve a win–win situation among channel members when manufacturers bear CSR. In terms of supply chain decision making, Song et al. [20] studied the problem of considering CSR in a supply chain consisting of one supplier and two retailers to analyze the effect of uniform and differential pricing on the equilibrium solution. Wang et al. [21] incorporated both competition among retailers and CSR investment efficiency of supply chain members into the decision model and found that only through CSR cooperation among member firms could they achieve greater market share and higher system profits. 

Different power structures among supply chain member firms abound in real life. For example, in the home appliance manufacturing industry, there are both manufacturer-dominated power structures, such as Lenovo and Haier, and retailer-dominated power structures, such as Suning and Gome [22], as well as power structures where both parties are under equal statuses, such as Haier and Gome. Different power structures produce different decision-making behaviors; in a master–slave game, the dominant leader makes the decision first, followed by the follower, while in a Nash equilibrium game, both sides of the game are on equal footing and make decisions simultaneously. In general, the company with greater channel power in the supply chain can become the leader and then make favorable strategic decisions through the first-mover advantage it holds [23]. Ma et al. [24] found that there is indeed a difference between the level of advertising input of retailers and the level of quality input of manufacturers under different power structures. Gang et al. [25] and Maiti and Giri [26] have discussed the impact of channel power structure on product quality, etc., in supply chain studies, 

The above research only considers the green supply chain, power structures, and CSR separately. Recently, some scholars have started to combined CSR with different power structures. Ni et al. [27] explored the CSR allocation in supply chains under different power structures and found that regardless of the channel power structure, a reasonable CSR allocation can continuously improve the system’s overall benefits. Yang et al. [28] found that based on suppliers’ social responsibility aspects considering commodity safety under different power structures, the introduction of shared ex post contracts and cost-sharing ex ante contracts could enable manufacturers and retailers to fulfill their CSR better. Fan et al. [23] studied the issue of firms’ undertaking CSR on product quality decisions and their corresponding profitability under different power structures. 

There also have been some recent studies exploring CSR in the green supply chain. Biswas et al. [29] found that in a combination of four channel members performing green manufacturing and implementing CSR scenarios, retailers alone performing CSR was most beneficial to the overall supply chain performance. Furthermore, in terms of government subsidy efficiency, Li et al. [30] and Liu et al. [31] introduced government subsidies while considering corporate social responsibility and studied the impact of government subsidies on CSR and supply chain performance. Sang and Zhang [32] explored the impact of retailers’ corporate social responsibility on the decision-making of green supply chain members. Some empirical analyses have shown that socially responsible firms have more competitive advantages than firms that only consider economic profits [33]. 

Although the studies mentioned above have studied CSR of supply chain members from different perspectives, most of them only considered CSR of suppliers or retailers individually, and few studies have compared the impact difference of CSR of both parties. Besides, most of the studies take the traditional supply chain as the research object, and there are few studies involving CSR undertaken by green supply chain members. Furthermore, some of the studies considering CSR undertaken by green supply chain members only involved the green production of manufacturers and did not consider the green promotion of retailers, while in real life, joint green marketing activities of manufacturers and retailers have become a widespread and effective marketing method, so the research needs to be supplemented and improved. In Table 1, we have summarized some literatures that have strong relevance to this paper to further reveal the innovations and contributions of this paper. 

In summary, compared with the existing literature, the innovation of this paper is that, unlike previous studies that only considered the manufacturer’s unilateral green production decision, this paper introduced the socially responsible behavior of manufacturers and retailers into the study of joint green marketing decisions of green supply chain members in order to be closer to the actual situation, and explored the joint effect of different power structures and different socially responsible subjects on the green supply chain. The paper also explored the impact of the joint action of different power structures and different social responsibility holders on the manufacturer’s optimal product greenness and wholesale price decisions, the retailer’s optimal green marketing input and retail price decisions, the profit of both parties, and the overall profit of the supply chain. 

## 3. Problem Description and Assumptions

To simplify the model and focus the study, we only considered a two-tier green supply chain with one manufacturer and one retailer. The manufacturer was responsible for developing and producing a green product to reduce the environmental pollution of the product. At the same time, the retailer sold green products through a series of green marketing initiatives, i.e., the manufacturer and retailer engaged in joint green marketing activities.

To better promote the subsequent study, we first proposed some assumptions as follows:

**Assumption** **1.** *To further simplify the model without affecting the findings of the study, the fixed production cost of the manufacturer to produce a unit of a green product is neglected, but to improve the level of greenness of the product, the manufacturer still needs to invest in green R&D costs to improve the technology. Referring to Ghosh and Shah [33], let the manufacturer’s green R&D input cost be $C_{d}=\frac{1}{2}zg^{2}$, where* z>0 *is the green R&D cost coefficient, and* g *denotes the product’s greenness. *

**Assumption** **2.** *Consumers’ consumption awareness significantly affects the market demand for green products. If consumers’ green consumption awareness is weak, they will not be willing to pay a higher price than normal products for green products; then green products will be replaced by other normal products, and the research and development toward producing green products will be meaningless. Therefore, compared with that of the manufacturers in the green supply chain, the green promotion of retailers has irreplaceable significance in the sales of green products. In order to raise consumers’ awareness of green consumption and attract them to buy green products, retailers must invest in green marketing. Referring to Ghosh and Shah [34], let the green marketing input cost of retailers for green products be* $C_{a}=\frac{1}{2}bv^{2}$ *, where* b>0 *is the green promotion input cost coefficient, and* v *denotes the level of green promotion input of retailers. *

**Assumption** **3.** *The market demand for green products is influenced by the retail price, greenness level, and green marketing level, and consumers prefer to buy products with low price, high greenness, and high green marketing level. Assuming that the market demand function of products is a linear function, the market demand function can be obtained as* $q=a-p+k_{1}g+k_{2}v$ *, where* a>0 *denotes the potential market size of green products;* p *denotes the retail price of products;* $k_{1}>0$ *denotes the sensitivity coefficient of consumers to the greenness level of products; and* $k_{2}>0$ *denotes the sensitivity coefficient of consumers to the green marketing input level. *

Therefore, the profits of the manufacturer and the retailer at this point could be expressed as:

$$\pi_{m}=wq-C_{d}=w\left(a-p+k_{1}g+k_{2}v\right)-\frac{1}{2}zg^{2}\;\pi_{r}=(p-w)q-C_{a}=(p-w)(a-p+k_{1}g+k_{2}v)-\frac{1}{2}bv^{2}$$

At this time, in order to ensure the concavity of the objective functions of the manufacturer and the retailer so that the research has practical significance, it was necessary to satisfy $k_{1}^{2}b+k_{2}^{2}z<bz$; that is, limited to their respective green investment costs constraints, the manufacturer and retailer could not increase the market demand for green products by endlessly improving the level of the product’s green degree and green promotion. What needs special explanation is that the comparison and analysis section below were also conducted under this precondition.

**Assumption** **4.** *Drawing on Panda [15], when the manufacturer and the retailer have an awareness of social responsibility, their level of social responsibility (CSR) awareness is defined as the degree of concern for consumer surplus. According to the definition of consumer surplus, that it represents the difference between the actual price and the price that consumers are willing to pay when purchasing green products, the consumer surplus can be expressed as *$CS=\int_{p_{\min}}^{p_{\max}}qdp=\int_{p_{\min}}^{p_{\max}}(a-p+k_{1}g+k_{2}v)dp=\frac{1}{2}{\left(a-p+k_{1}g+k_{2}v\right)}^{2}$. * The degree of concern of both parties for consumer surplus is indicated by *φ*, where the larger the value of *φ*, the higher the degree of CSR undertaken by both parties, the stronger the sense of social responsibility, and conversely, the lower the value of, the weaker the sense of social responsibility. *

The major symbols involved in this paper are summarized in Table 2.

## 4. Model Construction and Solution

### 4.1. Decision Scenario with the Retailer Taking CSR

First of all, in the case of CSR undertaken by the retailer, it focuses simultaneously on its own profit and consumer surplus, i.e., makes decisions with the goal of maximizing its own utility. At this time, the manufacturer does not have CSR awareness, and it still makes decisions with the goal of maximizing its own profit. We solve the equilibrium strategy in the manufacturer-led decision scenario, retailer-led decision scenario, and Nash equilibrium decision scenario in turn. At this point, the utility functions of the manufacturer and the retailer are as follows:

(1) $$V_{m}=\pi_{m}=w\left(a-p+k_{1}g+k_{2}v\right)-\frac{1}{2}zg^{2}$$

(2) $$V_{r}=\pi_{r}+\varphi CS=(p-w)(a-p+k_{1}g+k_{2}v)-\frac{1}{2}bv^{2}+\frac{\varphi }{2}{\left(a-p+k_{1}g+k_{2}v\right)}^{2}$$

#### 4.1.1. Manufacturer-Led Decision Scenario

In a manufacturer-driven market, the manufacturer first determines the product’s green degree g and wholesale price w. The retailer then determines the retail price p and the green promotion v. The optimal equilibrium strategy, in this case, is obtained by using the inverse induction method, as shown in Proposition 1.

**Proposition** **1.**  *The optimal product green degree and wholesale price set by the manufacturer are as follows: *

$$g_{1}^{*}=\frac{abk_{1}}{2bz(2-\varphi )-(k_{1}^{2}b+2k_{2}^{2}z)},w_{1}^{*}=\frac{az(2b-b\varphi -k_{2}^{2})}{2bz(2-\varphi )-(k_{1}^{2}b+2k_{2}^{2}z)}\;$$

*The optimal product retail price and level of green promotion set by the retailer are as follows: *

$$p_{1}^{*}=\frac{az(3b-2b\varphi -k_{2}^{2})}{2bz(2-\varphi )-(k_{1}^{2}b+2k_{2}^{2}z)},\;v_{1}^{*}=\frac{ak_{2}z}{2bz(2-\varphi )-(k_{1}^{2}b+2k_{2}^{2}z)}\;$$

*The optimal profits of the manufacturer, the retailer and the whole supply chain are as follows: *

$$\pi_{m_{1}}^{*}=\frac{a^{2}bz}{4bz(2-\varphi )-2(k_{1}^{2}b+2k_{2}^{2}z)},\pi_{r_{1}}^{*}=\frac{a^{2}bz^{2}(2b-b\varphi -k_{2}^{2})}{2{\left[2bz(2-\varphi )-(k_{1}^{2}b+2k_{2}^{2}z)\right]}^{2}},\pi_{sc_{1}}^{*}=\frac{a^{2}bz[3bz(2-\varphi )-(bk_{1}^{2}+3k_{2}^{2}z)]}{2{\left[2bz(2-\varphi )-(k_{1}^{2}b+2k_{2}^{2}z)\right]}^{2}}\;$$

#### 4.1.2. Retailer-Led Decision Scenario

In a retailer-driven market, the retailer first determines the retail price p and the green marketing input v of the green product, and then the manufacturer determines the greenness level g and the wholesale price w of the product on this basis. To facilitate the solution, let p=w+x and x be the retailer’s markup, and bring p=w+x into Equation (1) and solve it by reverse induction to obtain each optimal equilibrium strategy in this case, as shown in Proposition 2.

**Proposition** **2.** *The optimal product retail price and level of green promotion set by the retailer are as follows: *

$$p_{2}^{*}=\frac{ab(3z-k_{1}^{2}-\varphi z)}{bz(4-\varphi )-(2k_{1}^{2}b+k_{2}^{2}z)},v_{2}^{*}=\frac{ak_{2}z}{bz(4-\varphi )-(2k_{1}^{2}b+k_{2}^{2}z)}\;$$

*The optimal product green degree and wholesale price set by the manufacturer are as follows: *

$$g_{2}^{*}=\frac{abk_{1}}{bz(4-\varphi )-(2k_{1}^{2}b+k_{2}^{2}z)},w_{2}^{*}=\frac{abz}{bz(4-\varphi )-(2k_{1}^{2}b+k_{2}^{2}z)}\;$$

*The optimal profits of the manufacturer, the retailer and the whole supply chain are as follows: *

$$\pi_{m_{2}}^{*}=\frac{a^{2}b^{2}z(2z-k_{1}^{2})}{2{\left[bz(4-\varphi )-(2k_{1}^{2}b+k_{2}^{2}z)\right]}^{2}},\pi_{r_{2}}^{*}=\frac{a^{2}bz}{2[bz(4-\varphi )-(2k_{1}^{2}b+k_{2}^{2}z)]},\pi_{sc_{2}}^{*}=\frac{a^{2}bz[bz(6-\varphi )-(3bk_{1}^{2}+k_{2}^{2}z)]}{2{\left[bz(4-\varphi )-(2k_{1}^{2}b+k_{2}^{2}z)\right]}^{2}}\;$$

#### 4.1.3. Nash Equilibrium Decision Scenario

In a market where neither the retailer nor the manufacturer is the leader, the manufacturer and retailer make decisions simultaneously, with the retailer determining the retail price of the product p and the green marketing input v and the manufacturer determining the level of greenness of the product g and the wholesale price w, as shown in Proposition 3.

**Proposition** **3.** *The optimal product retail price and level of green promotion set by the retailer are as follows: *

$$p_{3}^{*}=\frac{abz(2-\varphi )}{bz(3-\varphi )-(k_{1}^{2}b+k_{2}^{2}z)},v_{3}^{*}=\frac{ak_{2}z}{bz(3-\varphi )-(k_{1}^{2}b+k_{2}^{2}z)}\;$$

*The optimal product green degree and wholesale price set by the manufacturer are as follows: *

$$g_{3}^{*}=\frac{abk_{1}}{bz(3-\varphi )-(k_{1}^{2}b+k_{2}^{2}z)},w_{3}^{*}=\frac{abz}{bz(3-\varphi )-(k_{1}^{2}b+k_{2}^{2}z)}\;$$

*The optimal profits of the manufacturer, the retailer and the whole supply chain are as follows: *

$$\pi_{m_{3}}^{*}=\frac{a^{2}b^{2}z(2z-k_{1}^{2})}{2{\left[bz(3-\varphi )-(k_{1}^{2}b+k_{2}^{2}z)\right]}^{2}},\pi_{r_{3}}^{*}=\frac{a^{2}bz^{2}(2b-b\varphi -k_{2}^{2})}{2{\left[bz(3-\varphi )-(k_{1}^{2}b+k_{2}^{2}z)\right]}^{2}}\;\pi_{sc_{3}}^{*}=\frac{a^{2}bz[bz(4-\varphi )-(bk_{1}^{2}+k_{2}^{2}z)]}{2{\left[bz(3-\varphi )-(k_{1}^{2}b+k_{2}^{2}z)\right]}^{2}}\;$$

### 4.2. Decision Scenario with the Manufacturer Taking CSR

Similarly, when the manufacturer takes CSR, it pays attention to consumer surplus while paying attention to its own profit, i.e., it makes decisions with the goal of maximizing its own utility. In this case, the retailer does not have a sense of CSR, and it still makes decisions with the goal of maximizing its own profit. We also solve the equilibrium strategies under the manufacturer-led decision scenario, retailer-led decision scenario, and Nash equilibrium decision scenario, successively. At this point, the utility functions of the manufacturer and the retailer are as follows:

(3) $$V_{m}=\pi_{m}+\varphi CS=w\left(a-p+k_{1}g+k_{2}v\right)-\frac{1}{2}zg^{2}+\frac{\varphi }{2}{\left(a-p+k_{1}g+k_{2}v\right)}^{2}$$

(4) $$V_{r}=\pi_{r}=(p-w)(a-p+k_{1}g+k_{2}v)-\frac{1}{2}bv^{2}$$

#### 4.2.1. Manufacturer-Led Decision Scenario

In a manufacturer-driven market, the manufacturer first determines the greenness level g and wholesale price w of the green product, and then the retailer decides the retail price p of the product and the green marketing input v on this basis. Using the inverse induction method, each equilibrium result can be obtained, as shown in Proposition 4.

**Proposition** **4.** *The optimal product green degree and wholesale price set by the manufacturer are as follows: *

$$g_{1}^{**}=\frac{abk_{1}}{bz(4-\varphi )-(k_{1}^{2}b+2k_{2}^{2}z)},w_{1}^{**}=\frac{az(2b-b\varphi -k_{2}^{2})}{bz(4-\varphi )-(k_{1}^{2}b+2k_{2}^{2}z)}\;$$

*The optimal product retail price and level of green promotion set by the retailer are as follows: *

$$p_{1}^{**}=\frac{az(3b-b\varphi -k_{2}^{2})}{bz(4-\varphi )-(k_{1}^{2}b+2k_{2}^{2}z)},v_{1}^{**}=\frac{ak_{2}z}{bz(4-\varphi )-(k_{1}^{2}b+2k_{2}^{2}z)}\;$$

*The optimal profits of the manufacturer, the retailer and the whole supply chain are as follows: *

$$\pi_{m_{1}}^{**}=\frac{a^{2}bz}{2bz(4-\varphi )-2(k_{1}^{2}b+2k_{2}^{2}z)},\pi_{r_{1}}^{**}=\frac{a^{2}bz^{2}(2b-k_{2}^{2})}{2{\left[bz(4-\varphi )-(k_{1}^{2}b+2k_{2}^{2}z)\right]}^{2}},\pi_{sc_{1}}^{**}=\frac{a^{2}bz[bz(6-\varphi )-(bk_{1}^{2}+3k_{2}^{2}z)]}{2{\left[bz(4-\varphi )-(k_{1}^{2}b+2k_{2}^{2}z)\right]}^{2}}\;$$

#### 4.2.2. Retailer-Led Decision Scenario

In a retailer-driven market, the retailer first determines the retail price of the product p and the green marketing input v. The manufacturer then determines the greenness of the product g and the wholesale price w on this basis. For ease of solution, let p=w+x and x be the retailer’s markup, and bring p=w+x into Equation (1) to obtain the manufacturer’s utility function as:

$$V_{m}=w\left(a-w-x+k_{1}g+k_{2}v\right)-\frac{1}{2}zg^{2}+\frac{\varphi }{2}{\left(a-w-x+k_{1}g+k_{2}v\right)}^{2}$$

Using the reverse induction method, the optimal equilibrium strategies in this case can be obtained, as shown in Proposition 5.

**Proposition** **5.** *The optimal product retail price and level of green promotion set by the retailer are as follows: *

$$p_{2}^{**}=\frac{ab(3z-k_{1}^{2}-2\varphi z)}{2bz(2-\varphi )-(2k_{1}^{2}b+k_{2}^{2}z)},v_{2}^{**}=\frac{ak_{2}z}{2bz(2-\varphi )-(2k_{1}^{2}b+k_{2}^{2}z)}\;$$

*The optimal product green degree and wholesale price set by the manufacturer are as follows: *

$$g_{2}^{**}=\frac{abk_{1}}{2bz(2-\varphi )-(2k_{1}^{2}b+k_{2}^{2}z)},w_{2}^{**}=\frac{abz(1-\varphi )}{2bz(2-\varphi )-(2k_{1}^{2}b+k_{2}^{2}z)}\;$$

*The optimal profits of the manufacturer, the retailer and the whole supply chain are as follows: *

$$\pi_{m_{2}}^{**}=\frac{a^{2}b^{2}z(2z-k_{1}^{2}-z\varphi )}{2{\left[2bz(2-\varphi )-(2k_{1}^{2}b+k_{2}^{2}z)\right]}^{2}},\pi_{r_{2}}^{**}=\frac{a^{2}bz}{2[2bz(2-\varphi )-(2k_{1}^{2}b+k_{2}^{2}z)]},\pi_{sc_{2}}^{**}=\frac{a^{2}bz[3bz(2-\varphi )-(3bk_{1}^{2}+k_{2}^{2}z)]}{2{\left[2bz(2-\varphi )-(2k_{1}^{2}b+k_{2}^{2}z)\right]}^{2}}\;$$

#### 4.2.3. Nash Equilibrium Decision Scenario

In a market where neither the retailer nor the manufacturer is the leader, the manufacturer and retailer make decisions simultaneously, with the retailer determining the retail price of the product p and the green marketing input v and the manufacturer determining the level of greenness of the product g and the wholesale price w, as shown in Proposition 6.

**Proposition** **6.** *The optimal product retail price and level of green promotion set by the retailer are as follows: *

$$p_{3}^{**}=\frac{abz(2-\varphi )}{bz(3-\varphi )-(k_{1}^{2}b+k_{2}^{2}z)},v_{3}^{**}=\frac{ak_{2}z}{bz(3-\varphi )-(k_{1}^{2}b+k_{2}^{2}z)}\;$$

*The optimal product green degree and wholesale price set by the manufacturer are as follows: *

$$g_{3}^{**}=\frac{abk_{1}}{bz(3-\varphi )-(k_{1}^{2}b+k_{2}^{2}z)},w_{3}^{**}=\frac{abz(1-\varphi )}{bz(3-\varphi )-(k_{1}^{2}b+k_{2}^{2}z)}\;$$

*The optimal profits of the manufacturer, the retailer and the whole supply chain are as follows: *

$$\pi_{m_{3}}^{**}=\frac{a^{2}b^{2}z(2z-k_{1}^{2}-z\varphi )}{2{\left[bz(3-\varphi )-(k_{1}^{2}b+k_{2}^{2}z)\right]}^{2}},\pi_{r_{3}}^{**}=\frac{a^{2}bz^{2}(2b-k_{2}^{2})}{2{\left[bz(3-\varphi )-(k_{1}^{2}b+k_{2}^{2}z)\right]}^{2}},\pi_{sc_{3}}^{**}=\frac{a^{2}bz[bz(4-\varphi )-(bk_{1}^{2}+k_{2}^{2}z)]}{2{\left[bz(3-\varphi )-(k_{1}^{2}b+k_{2}^{2}z)\right]}^{2}}\;$$

## 5. Comparison and Analysis

Since there are many decision scenarios involved, in order to show more clearly and comprehensively the effects of different CSR bearers and different power structures on each equilibrium strategies of the manufacturer and retailer, this section first explores the effects of different power structures on each equilibrium solution under two decision scenarios, namely, CSR taken by the retailer and CSR taken by the manufacturer, and then explores the effects of different CSR bearers on each equilibrium solution under three decision scenarios, namely, manufacturer-led, retailer-led, and Nash equilibrium.

### 5.1. Analysis of the Impact of Different Power Structures on the Equilibrium Results in Terms of the Same CSR Bearer

#### 5.1.1. CSR Taken by the Retailer

**Corollary** **1.** $\frac{\partial g_{j}^{*}}{\partial \varphi }>0$*, *$\frac{\partial w_{j}^{*}}{\partial \varphi }>0$*, *$\frac{\partial v_{j}^{*}}{\partial \varphi }>0$*, *$\frac{\partial \pi_{m_{j}}^{*}}{\partial \varphi }>0$*, *$\frac{\partial \pi_{r_{j}}^{*}}{\partial \varphi }>0$*, *$\frac{\partial \pi_{sc_{j}}^{*}}{\partial \varphi }>0$*, *$\frac{\partial p_{j}^{*}}{\partial \varphi }<0$(j=1,2,3).

Corollary 1 shows that no matter under which kind of power structure, the product green degree, the wholesale price, the green promotion investment, the manufacturer’s profit, the retailer’s profit, and the whole supply chain’s profit are all positively related to the intensity of the retailer’s awareness of CSR. In contrast, the product’s retail price is negatively related to φ. This indicates that the stronger the retailer’s awareness of CSR, the higher the product green degree, wholesale price, manufacturer’s profit, retailer’s profit, and total profit of the supply chain are, while the retail price is lower. This is because when the retailer has a sense of CSR, it chooses to reduce retail price and increase investment in green promotion out of special attention to consumer surplus. At the same time, affected by the retailer’s awareness of CSR, the manufacturer chooses to increase the product’s green degree and further increase the wholesale price to maintain its own profit. In addition, the above phenomenon also shows that regardless of the power structure, it is beneficial for all members of the supply chain and the entire supply chain when retailers take CSR. The proofs are shown in Appendix A.

**Corollary** **2.** *When *$0<\varphi \leq \frac{b-k_{2}^{2}}{b}$*, *$w_{1}^{*}\geq w_{3}^{*}>w_{2}^{*}$, $p_{2}^{*}>p_{1}^{*}\geq p_{3}^{*}$; when $\frac{b-k_{2}^{2}}{b}<\varphi <1$*, *$w_{3}^{*}>w_{1}^{*}>w_{2}^{*}$, $p_{2}^{*}>p_{3}^{*}>p_{1}^{*}$.

Corollary 2 shows that the wholesale price is lowest and the retail price is highest when the retailer dominates the supply chain compared to the other two power structures. This is because when the retailer dominates the supply chain, it has more substantial bargaining power, and in order to obtain as much marginal revenue as possible, it controls the wholesale price to the lowest level possible and adjusts the retail price to the highest level possible. However, as the retailer’s awareness of CSR gradually increases, the wholesale price increases more rapidly when both parties’ power levels are equal than in the other two power structures, and the retail price decreases more rapidly under the manufacturer-led scenario than under the Nash equilibrium scenario. Because the retail price is negatively correlated with the retailer’s CSR awareness intensity, the retail price reaches the lowest level under the manufacturer-led scenario after a certain CSR level is reached. The proofs are shown in Appendix B.

**Corollary** **3.** *When *$\frac{b-k_{2}^{2}}{b}\leq \varphi <1$*, *$g_{1}^{*}\geq g_{3}^{*}>g_{2}^{*}$, $v_{1}^{*}\geq v_{3}^{*}>v_{2}^{*}$; when $k_{1}^{2}b>k_{2}^{2}z$*and *$0<\varphi \leq \frac{k_{1}^{2}b-k_{2}^{2}z}{bz}$*, *$g_{3}^{*}>g_{2}^{*}\geq g_{1}^{*}$, $v_{3}^{*}>v_{2}^{*}\geq v_{1}^{*}$; when $k_{1}^{2}b>k_{2}^{2}z$*and *$\frac{k_{1}^{2}b-k_{2}^{2}z}{bz}<\varphi <\frac{b-k_{2}^{2}}{b}$*, *$g_{3}^{*}>g_{1}^{*}>g_{2}^{*}$, $v_{3}^{*}>v_{1}^{*}>v_{2}^{*}$.

Corollary 3 shows that the relationship of the product green degree and green promotion among the three power structures is significantly influenced by the intensity of CSR taken by the retailer. When $0<\varphi <\frac{b-k_{2}^{2}}{b}$, the product green degree and green promotion input are always the highest under the Nash equilibrium scenario. This indicates that when both parties’ power levels are equal, the competition between the manufacturer and the retailer intensifies, prompting the manufacturer to improve the level of product green degree and the retailer to improve the level of green promotion input. As the retailer’s awareness of CSR gradually increases, the product green degree and green promotion input grow the fastest under the manufacturer-led scenario and eventually exceed those under the Nash equilibrium scenario.

**Corollary** **4.** $\pi_{m_{1}}^{*}>\pi_{m_{3}}^{*}>\pi_{m_{2}}^{*}$; when $0<\varphi <\frac{b-k_{2}^{2}}{b}$*, *$\pi_{r_{2}}^{*}>\pi_{r_{3}}^{*}>\pi_{r_{1}}^{*}$; when $\frac{b-k_{2}^{2}}{b}\leq \varphi <1$*, *$\pi_{r_{2}}^{*}>\pi_{r_{1}}^{*}\geq \pi_{r_{3}}^{*}$.

Corollary 4 shows that the manufacturer’s profit is the largest when the manufacturer dominates the supply chain and the smallest when the retailer dominates the supply chain. It is easy to understand that this is because the manufacturer has the greatest power when it dominates the supply chain to obtain higher profit for itself through a series of measures. In contrast, when the retailer dominates the supply chain, the retailer has the greatest power, so the manufacturer’s profit is inevitably squeezed. Similarly, the retailer’s profit is highest when it dominates the supply chain and grows fastest when the manufacturer dominates the supply chain as the retailer’s awareness of CSR gradually increases. This is because the product green degree grows faster for consumers when the manufacturer dominates the supply chain, while the retail price is lower in that case, thus attracting more consumers to purchase green products and ultimately leading to a higher level of profit for the retailer.

#### 5.1.2. CSR Taken by the Manufacturer

**Corollary** **5.** $\frac{\partial g_{j}^{*}}{\partial \varphi }>0$*, *$\frac{\partial v_{j}^{*}}{\partial \varphi }>0$*, *$\frac{\partial \pi_{m_{j}}^{*}}{\partial \varphi }>0$*, *$\frac{\partial \pi_{r_{j}}^{*}}{\partial \varphi }>0$*, *$\frac{\partial \pi_{sc_{j}}^{*}}{\partial \varphi }>0$*, *$\frac{\partial p_{j}^{*}}{\partial \varphi }<0$*, *$\frac{\partial w_{j}^{*}}{\partial \varphi }<0$*, *(j=1,2,3).

Corollary 5 shows that no matter under which kind of power structure, the product green degree, the green promotion investment, the manufacturer’s profit, the retailer’s profit, and the whole supply chain’s profit are all positively related to the intensity of the manufacturer’s awareness of CSR, while both the retail price and the wholesale price are negatively related to φ. This suggests that the stronger the manufacturer’s awareness of CSR, the higher the product green degree, the manufacturer’s profit, the retailer’s profit, and the total profit of the supply chain are, and the lower the retail price and wholesale price are. This is because when the manufacturer has a sense of CSR, as in the case when the retailer has a sense of CSR, it chooses to reduce the wholesale price and improve the product green degree out of special attention to consumer surplus. At the same time, affected by the manufacturer’s awareness of CSR, the retailer chooses to increase investment in product green promotion and further reduce the retail price. In addition, the above phenomenon also shows that regardless of the power structure, it is beneficial for all members of the supply chain and the entire supply chain when manufacturers take CSR. The proofs are shown in Appendix C.

**Corollary** **6.** *The optimal wholesale price under the three power structures satisfies: *$w_{1}^{**}>w_{3}^{**}>w_{2}^{**}$.

Corollary 6 shows that the product wholesale price is the highest when the manufacturer dominates the supply chain, the second-highest when both parties jointly dominate the supply chain, and the lowest when the retailer dominates the supply chain. This is because when the manufacturer dominates the supply chain, it has more substantial bargaining power and can control the whole supply chain, so it tries its best to obtain a higher marginal revenue by setting the wholesale price to a higher level.

**Corollary** **7.** *When *$\frac{z-k_{1}^{2}}{z}\leq \varphi <1$*, *$g_{2}^{**}\geq g_{3}^{**}>g_{1}^{**}$, $v_{2}^{**}\geq v_{3}^{**}>v_{1}^{**}$, $p_{1}^{**}>p_{3}^{**}>p_{2}^{**}$; when $k_{2}^{2}z<k_{1}^{2}b$*and *$0<\varphi <\frac{z-k_{1}^{2}}{z}$*, *$g_{3}^{**}>g_{2}^{**}>g_{1}^{**}$, $v_{3}^{**}>v_{2}^{**}>v_{1}^{**}$, $p_{1}^{**}>p_{2}^{**}>p_{3}^{**}$; when $k_{2}^{2}z>k_{1}^{2}b$*and *$0<\varphi <\frac{k_{2}^{2}z-k_{1}^{2}b}{bz}$*, *$g_{3}^{**}>g_{1}^{**}>g_{2}^{**}$, $v_{3}^{**}>v_{1}^{**}>v_{2}^{**}$, $p_{2}^{**}\geq p_{1}^{**}>p_{3}^{**}$; when $k_{2}^{2}z>k_{1}^{2}b$*and *$\frac{k_{1}^{2}b-k_{2}^{2}z}{bz}\leq \varphi <\frac{z-k_{1}^{2}}{z}$*, *$g_{3}^{**}>g_{2}^{**}\geq g_{1}^{**}$, $v_{3}^{**}>v_{2}^{**}\geq v_{1}^{**}$, $p_{1}^{**}>p_{2}^{**}>p_{3}^{**}$.

Corollary 7 shows that the relationship among the product green degree, the green promotion input, and retail price under the three power structures is significantly influenced by the intensity of CSR taken by the manufacturer. When $0<\varphi <\frac{z-k_{1}^{2}}{z}$, the product green degree and the green promotion input are both always the highest under the Nash equilibrium scenario, while the retail price is always the lowest. This indicates that as in the case when the retailer has a sense of CSR, the situation where both parties dominate the supply chain intensifies the competition between the manufacturer and the retailer, prompting the manufacturer to increase the level of the product green degree and the retailer to increase its green promotion investment. From the consumer’s perspective, the situation where both parties dominate the supply chain is the best compared with the other two power structures, because the retail price of the product is the lowest while the product green degree is the highest. However, as the manufacturer’s awareness of CSR gradually increases, the case where the retailer dominates the supply chain has the fastest growth rate in product green degree and green promotion investment, but the fastest decrease rate in the retail price, eventually exceeding the case when both parties jointly dominate the supply chain.

**Corollary** **8.** *When *$0<\varphi <\frac{z-k_{1}^{2}}{z}$*, *$\pi_{m_{1}}^{**}>\pi_{m_{3}}^{**}>\pi_{m_{2}}^{**}$; when $\frac{z-k_{1}^{2}}{z}\leq \varphi <1$*, *$\pi_{m_{1}}^{**}>\pi_{m_{2}}^{**}\geq \pi_{m_{3}}^{**}$; $\pi_{r_{2}}^{**}>\pi_{r_{3}}^{**}>\pi_{r_{1}}^{**}$.

Corollary 8 shows that the manufacturer’s profit is the highest when it dominates the supply chain and grows the fastest when the retailer dominates the supply chain as the manufacturer’s awareness of CSR gradually increases. This is because the product demand function is negatively related to the retail price and positively related to the green degree and green promotion investment. Under the retailer-led scenario, when the potential market demand a is specific, the retail price of the product is the lowest, so the market demand is the highest, which is conducive to the manufacturer expanding sales and increasing its profit.

Similarly, when the manufacturer takes CSR, the retailer’s profit is the highest when the retailer dominates the supply chain, the second-highest when the retailer and the manufacturer jointly dominate the supply chain, and the lowest when the manufacturer dominates the supply chain. This is because when the retailer dominates the supply chain, its bargaining power is the largest in the whole supply chain, so it can increase its profit as much as possible in many ways. When both parties jointly dominate the supply chain, the power levels of both parties are equivalent, while when the manufacturer dominates the supply chain, the retailer has the least power, so the retailer’s profit is squeezed, resulting in a lower level of profit.

### 5.2. Analysis of the Impact of Different CSR Bearers on the Equilibrium Results in Terms of the Same Power Structure

**Corollary** **9.** *When the manufacturer dominates the supply chain, there exists: (1) *$g_{1}^{*}>g_{1}^{**}$; (2) $w_{1}^{*}>w_{1}^{**}$; (3) $p_{1}^{*}<p_{1}^{**}$; (4) $v_{1}^{*}>v_{1}^{**}$; (5) $\pi_{m_{1}}^{*}>\pi_{m_{1}}^{**}$; (6) $\pi_{r_{1}}^{*}>\pi_{r_{1}}^{**}$.

Corollary 9 shows that when the manufacturer dominates the supply chain, the product green degree, the wholesale price, green promotion investment, the manufacturer’s profit, and the retailer’s profit are all higher when the retailer takes CSR compared to the case where the manufacturer takes CSR itself, while the retail price is lower. In addition, this also shows that when the manufacturer is the leader of the supply chain, it should actively encourage the retailer to take CSR rather than taking CSR itself, because this is not only beneficial to itself, but also beneficial to the retailer.

**Corollary** **10.** *When the retailer dominates the supply chain, there exists: (1)*$g_{2}^{*}<g_{2}^{**}$; (2) $w_{2}^{*}>w_{2}^{**}$; (3)$p_{r_{2}}^{*}>p_{r_{2}}^{**}$; (4)$v_{2}^{*}<v_{2}^{**}$; (5)$\pi_{m_{2}}^{*}<\pi_{m_{2}}^{**}$; (6)$\pi_{r_{2}}^{*}<\pi_{r_{2}}^{**}$.

Corollary 10 shows that when the retailer dominates the supply chain, the product green degree, the green promotion investment, the manufacturer’s profit, and the retailer’s profit are lower, while the wholesale price and retail price are higher when it takes CSR compared to the case where the manufacturer takes CSR. The above phenomenon shows that when the retailer is the leader of the supply chain, it should actively encourage the manufacturer to take CSR rather than taking CSR itself, because this is not only beneficial to itself, but also beneficial to the manufacturer.

**Corollary** **11.** *When both parties jointly dominate the supply chain, there exists: (1)*$w_{3}^{*}>w_{3}^{**}$, $g_{3}^{*}{=g}_{3}^{**}$, $p_{3}^{*}=p_{3}^{**}$, $v_{3}^{*}=v_{3}^{**}$; (2)$\pi_{m_{3}}^{*}>\pi_{m_{3}}^{**}$, $\pi_{r_{3}}^{*}<\pi_{r_{3}}^{**}$, $\pi_{SC_{3}}^{*}=\pi_{SC_{3}}^{**}$.

Corollary 11 (1) shows that when the retailer and the manufacturer jointly dominate the supply chain, the wholesale price of the product is higher when the retailer takes CSR compared to the case where the manufacturer takes CSR. This is because when the retailer takes CSR and the manufacturer does not take CSR, the manufacturer does not have to consider consumer surplus, so it further increases its marginal revenue by raising the wholesale price. The product green degree, the retail price, and the green promotion investment are equal whether the manufacturer or the retailer takes CSR.

Corollary 11 (2) shows that when the retailer and the manufacturer jointly dominate the market, the manufacturer’s profit is larger, but the retailer’s profit is smaller, when the retailer takes CSR compared to the case where the manufacturer takes CSR, while the total profit of the supply chain is equal. The above phenomenon shows that in the green supply chain, when the power of manufacturers and retailers is equal, for the sake of their own profits, both manufacturers and retailers should encourage the other party to take CSR, but from the perspective of the overall profit of the supply chain, which party takes CSR is irrelevant.

## 6. Numerical Analysis

In this section, considering the complexity of the equilibrium solution, in order to clearly demonstrate the effects of different power structures and the different degrees of CSR assumed by manufacturers and retailers on each equilibrium outcome and to gain some valuable management insights, we used numerical simulation analysis to verify the conclusions obtained in the previous section. The relevant parameters were set to a=200, b=20, z=20, $k_{1}=1$, and $k_{2}=1$ by referring to the relevant literature and combining it with the assumptions in the previous section of this paper:

(1) The impact of the intensity of retailer’s awareness of CSR *φ * on equilibrium results under different power structures.
(2) The impact of the intensity of manufacturer’s awareness of CSR on equilibrium results under different power structures.
(3) The impact of the intensity of retailer’s and manufacturer’s awareness of CSR *φ * on equilibrium results when the manufacturer dominates the supply chain.
(4) The impact of the intensity of retailer’s and manufacturer’s awareness of CSR *φ * on equilibrium results when the retailer dominates the supply chain.

**Figure 1:**
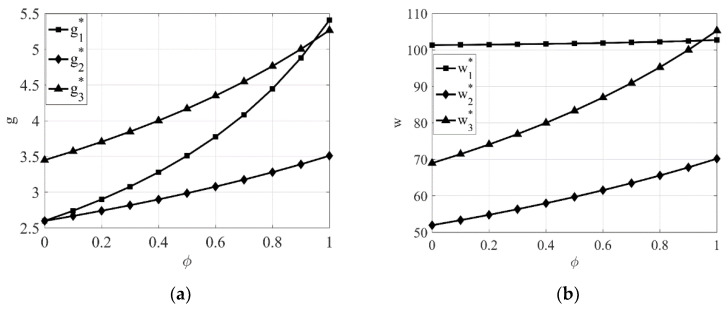

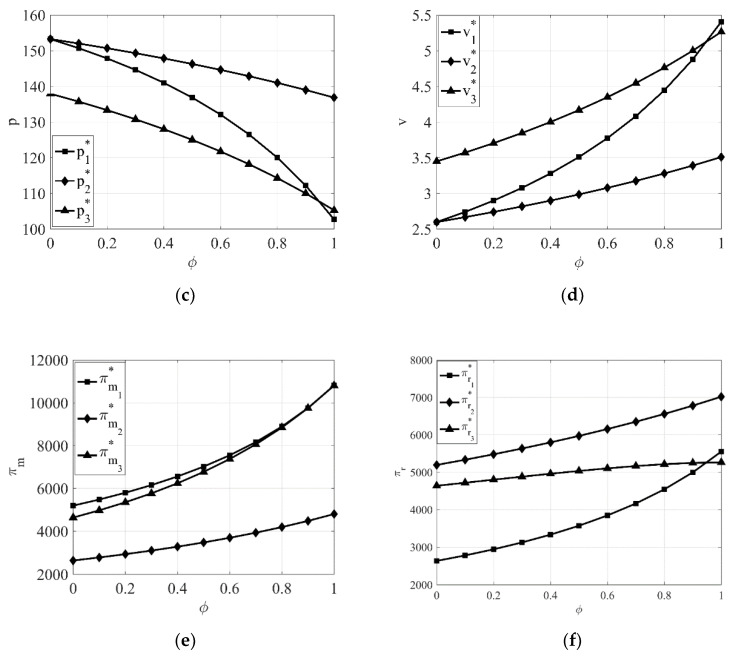
The impact of the intensity of retailer’s awareness of CSR φ on equilibrium results under different power structures. (**a**) The impact of φ on product green degree. (**b**) The impact of φ on wholesale price. (**c**) The impact of φ on retail price. (**d**) The impact of φ on green promotion. (**e**) The impact of φ on the manufacturer’s profit. (**f**) The impact of φ on the retailer’s profit.

From Figure 1a–f, it can be seen that regardless of the power structure, the product green degree, the wholesale price, the green promotion input, the manufacturer’s profit, and the retailer’s profit all increase as the retailer’s awareness of CSR gradually increases, while the retail price decreases. This further validates the conclusions of Corollary 1. In addition, we can clearly see from Figure 1a,d that with the continuous enhancement of the retailer’s awareness of CSR, the relationship of product green degree under the three power structures gradually changes from $g_{3}^{*}>g_{1}^{*}>g_{2}^{*}$ to $g_{1}^{*}\geq g_{3}^{*}>g_{2}^{*}$, and the relationship of green promotion under the three power structures gradually changes to $v_{3}^{*}>v_{1}^{*}>v_{2}^{*}$. This indicates that as the retailer’s awareness of CSR gradually increases, the growth rates of product green degree and green promotion investment are greatest when the manufacturer dominates the supply chain, followed by the retailer and the manufacturer co-dominating the supply chain, and the smallest when the retailer dominates the supply chain. This also further validates the conclusions of Corollary 3.

**Figure 2:**
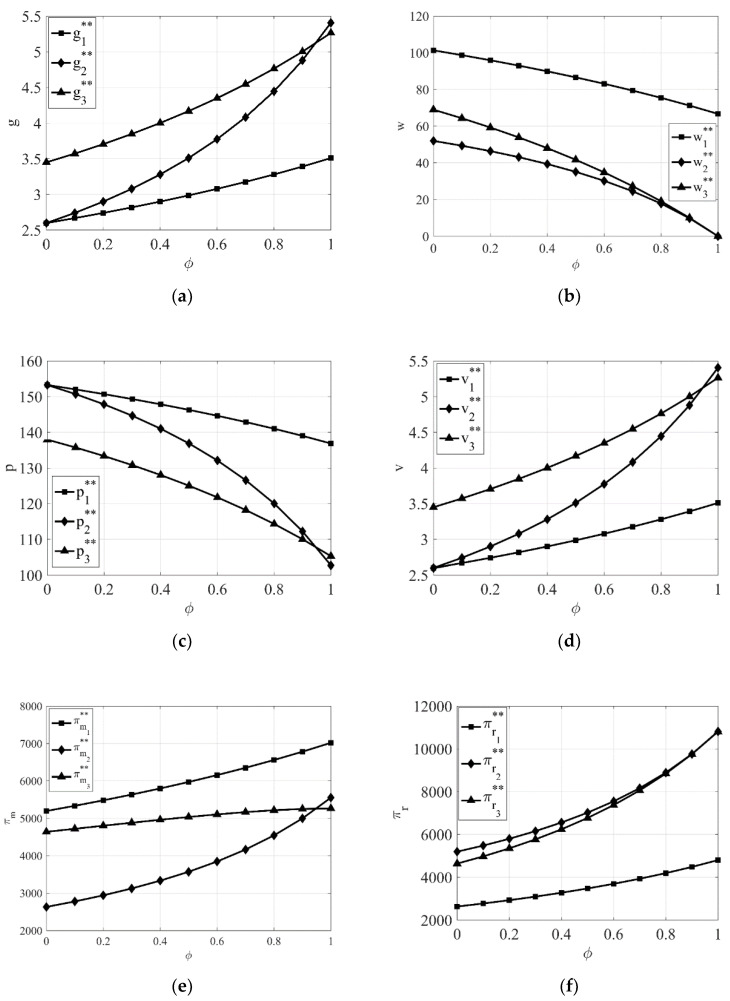
The impact of the intensity of manufacturer’s awareness of CSR φ on equilibrium results under different power structures. (**a**) The impact of φ on product green degree. (**b**) The impact of φ on wholesale price. (**c**) The impact of φ on retail price. (**d**) The impact of φ on green promotion. (**e**) The impact of φ on the manufacturer’s profit. (**f**) The impact of φ on the retailer’s profit.

From Figure 2a–f, it can be seen that regardless of the power structure, the product green degree, green promotion investment, manufacturer’s profit, and retailer’s profit all increase as the manufacturer’s awareness of CSR gradually increases, while the retail price and wholesale price both decrease. This further validates the conclusions of Corollary 5. In addition, we can also clearly see from Figure 2a,d that with the continuous enhancement of the manufacturer’s awareness of CSR, the relationship of product green degree under the three power structures gradually changes from $g_{3}^{**}>g_{2}^{**}>g_{1}^{**}$ to $g_{2}^{**}\geq g_{3}^{**}>g_{1}^{**}$, and the relationship of green promotion under the three power structures also gradually changes from $v_{3}^{**}>v_{2}^{**}>v_{1}^{**}$ to $v_{2}^{**}\geq v_{3}^{**}>v_{1}^{**}$. This indicates as the manufacturer’s awareness of CSR gradually increases, the growth rates of product green degree and green promotion investment are greatest when the retailer dominates the supply chain, followed by the retailer and the manufacturer co-dominating the supply chain, and the smallest when the manufacturer dominates the supply chain. This also further validates the conclusions of Corollary 7.

From Figure 1e and Figure 2e, it can be seen that the manufacturer’s profit is highest when the manufacturer itself dominates the supply chain compared with the other two power structures whether the manufacturer or the retailer takes CSR. Similarly, from Figure 1f and Figure 2f, it can be seen that the retailer’s profit is highest when the retailer itself dominates the supply chain compared with the other two power structures whether the manufacturer or the retailer takes CSR. This also shows once again that no matter which party takes CSR, the profit of one party is maximized when it dominates the supply chain.

**Figure 3:**
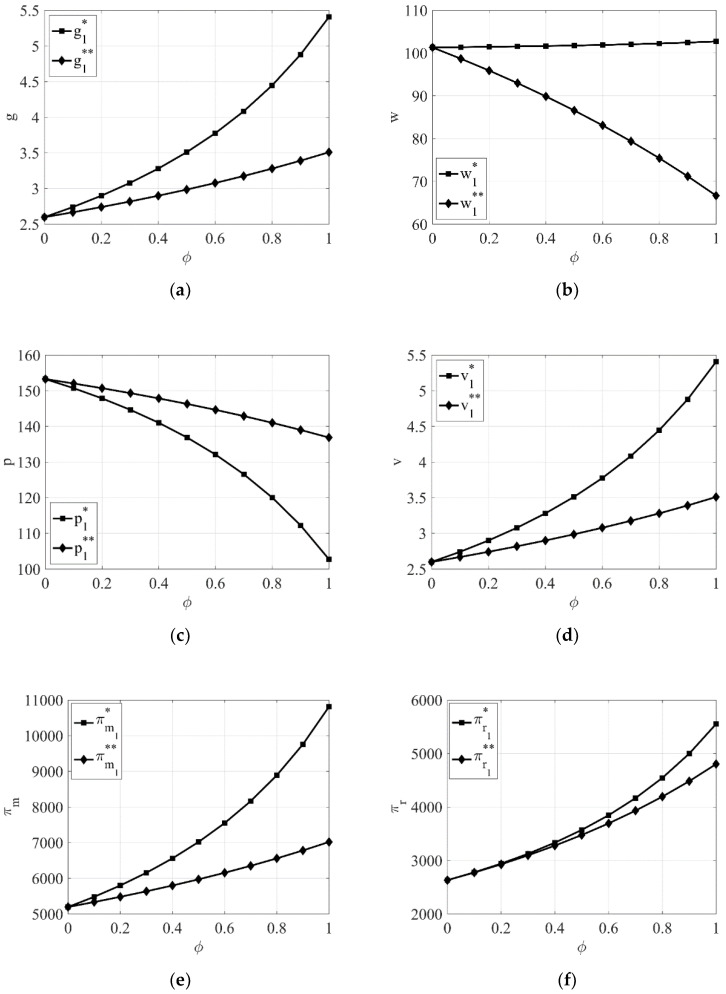
The impact of the intensity of the retailer’s and manufacturer’s awareness of CSR φ on equilibrium results when the manufacturer dominates the supply chain. (**a**) The impact of φ on product green degree. (**b**) The impact of φ on wholesale price. (**c**) The impact of φ on retail price. (**d**) The impact of φ on green promotion. (**e**) The impact of φ on the manufacturer’s profit. (**f**) The impact of φ on the retailer’s profit.

From Figure 3a,c–f, it can be seen that when the manufacturer dominates the supply chain, the product green degree, the green promotion investment, the manufacturer’s profit, and the retailer’s profit are higher, while the retail price is lower, when the retailer takes CSR compared to when the manufacturer takes CSR.

**Figure 4:**
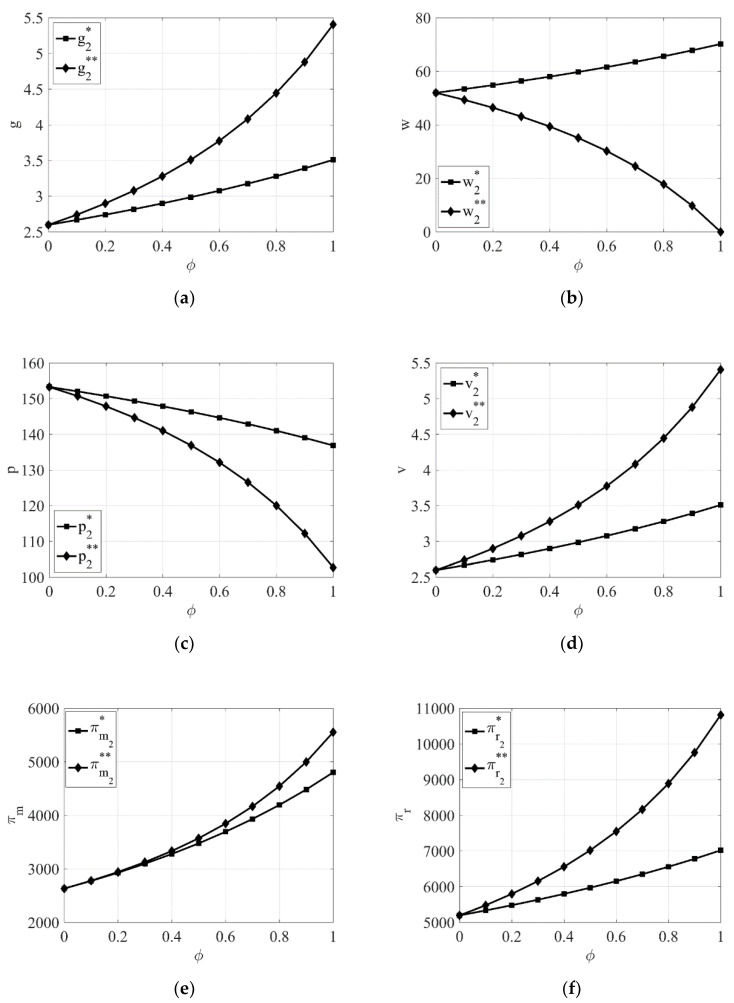
The impact of the intensity of the retailer’s and manufacturer’s awareness of CSR φ on equilibrium results when the retailer dominates the supply chain. (**a**) The impact of φ on product green degree. (**b**) The impact of φ on wholesale price. (**c**) The impact of φ on retail price. (**d**) The impact of φ on green promotion. (**e**) The impact of φ on the manufacturer’s profit. (**f**) The impact of φ on the retailer’s profit.

Moreover, from Figure 4a,c–f, it can also be seen that when the retailer dominates the supply chain, the product green degree, the green promotion investment, the manufacturer’s profit, and the retailer’s profit are all higher, while the retail price is lower, when the manufacturer takes CSR compared to when the retailer takes CSR.

From Figure 3b and Figure 4b, it can be seen that the wholesale price of green products is higher when the retailer takes CSR compared to when the manufacturer takes CSR regardless of the power structure.

## 7. Conclusions

### 7.1. Main Conclusions

This paper established three joint green marketing decision models for green supply chains under different power structures based on the cases of retailers or manufacturers taking up CSR and focusing on consumer surplus, and explored, in turn, the influence of different power structures on the optimal decisions of green supply chain members when manufacturers or retailers take up corporate social responsibility alone. It also explored the influence of the power structure when manufacturers or retailers take up CSR on the optimal decision-making of green supply chain members under the same power structure, and some meaningful conclusions were drawn through in-depth comparison and analysis. Mainly, the following conclusions were obtained:

(1) The ranking of the advantages and disadvantages of the three different power structures differs depending on the degree of CSR undertaken by manufacturers or retailers, which indicates that the ranking of the optimal equilibrium solutions under the three different power structures differs as the degree of social responsibility undertaken by enterprises increases. Regardless of the power structure, manufacturers and retailers actively undertake CSR to improve the level of the greenness of products and reduce the retail price of products, thus effectively promoting the increase of demand for green products in the market and ultimately increasing the profit level of manufacturers and retailers and the total profit level of the supply chain.
(2) Whether the manufacturer or the retailer bears CSR, the wholesale price of green products is higher when the manufacturer dominates the market than when the retailer dominates the market. This is because the manufacturer bears the R&D investment in improving the greenness of green products, and in order to compensate for the loss, the manufacturer sets a higher wholesale price to obtain as much revenue as possible when it dominates the market. This is because the retailer is concerned with consumer surplus and the manufacturer is not, so the manufacturer still raises its marginal revenue by increasing the wholesale price of the product.
(3) For manufacturers or retailers, whether they undertake CSR or not, to further improve their own profits, they should continuously improve their own strengths to achieve their own dominant market situations as much as possible. In addition, when manufacturers and retailers choose to undertake CSR, they should continue to increase their CSR commitment within their own capabilities because the more they undertake CSR, the better it will be for them and the more profits they will make. For consumers, it is advantageous for manufacturers or retailers in the green supply chain to take on CSR regardless of the power structure, because as manufacturers and retailers take on more CSR, the greenness of green products will increase while the retail price will decrease.

Based on the above conclusions, the following management insights can be obtained:

For manufacturers or retailers in the green supply chain, whether they undertake CSR or not, they should continuously improve their strengths and achieve their dominant positions as much as possible to further increase their profits. From the perspective of self-interest, when the manufacturer dominates the market, the manufacturer should encourage the retailer to take CSR. Similarly, when the retailer dominates the supply chain, the retailer should encourage the manufacturer to take CSR. When the retailer and the manufacturer jointly dominate the supply chain, both parties should choose to let the other party take CSR.

Both manufacturers and retailers in the green supply chain should actively undertake CSR within their own capacity because undertaking CSR is conducive to improving their own profits, to improving each other’s profits, and the profits of the whole supply chain, to providing higher green products to the market, and to increasing consumer surplus, thus further enhancing the overall competitive advantage of the green supply chain. Manufacturers and retailers should take CSR appropriately according to their own situations and determine the appropriate socially responsible investment according to their positions in the green supply chain. In addition, both parties should share the costs of CSR within their respective capabilities in order to share the benefits of CSR.

### 7.2. Research Limitations

Undeniably, there are still some shortcomings in this study that need to be explored in future research. On the one hand, this paper considered a linear demand function and did not consider the character of the existence of randomness in market demand. On the other hand, this paper only considered a secondary green supply chain consisting of a manufacturer and a retailer and did not consider the situation where the members of the green supply chain take on CSR in a complex market environment, and these issues deserve further research. In addition, since one of the purposes of this paper was to compare the impact of CSR undertaken by different members of the green supply chain, in order to avoid the coefficients of CSR undertaken by both parties appearing simultaneously in the relevant model, the case of CSR undertaken by both parties at the same time was not considered in this paper. Related issues need to be investigated in further research.

## Figures and Tables

**Table 1:** Some literature most relevant to this paper.

Author	Green Supply Chain	Power Structures	CSR	Joint Green Marketing
Ghosh and Shah [34]	✓	✓		
Hong and Guo [12]	✓	✓		
Sang and Zhang [32].	✓		✓	
Zhang et al. [5]	✓			
Zhou et al. [35]	✓			✓
This paper	✓	✓	✓	✓

**Table 2:** Definition of major symbols.

**Decision Variables**	**Definition**
$g_{j}^{u}$	Product greenness, j=1,2,3, u=*,**
$v_{j}^{u}$	Product green promotion, j=1,2,3, u=*,**
$w_{j}^{u}$	Wholesale price, j=1,2,3, u=*,**
$p_{j}^{u}$	Retail price, j=1,2,3, u=*,**
**Parameters**	**Definition**
a	Potential market demand for green products, a>0
$k_{1}$	Sensitivity coefficient of consumers to the product’s green degree, $k_{1}>0$
$k_{2}$	Sensitivity coefficient of consumers to the product’s green promotion, $k_{2}>0$
φ	The intensity of the manufacturer’s or retailer’s awareness of CSR, 0<φ<1
$\pi_{i_{j}}^{u}$	Profit for each decision maker, i=m,r,sc, j=1,2,3, u=*,**
$V_{s}^{u}$	Utility of each decision maker, s=m,r, u=*,**
z	Green R&D cost coefficient, z>0
b	Green promotion cost coefficient, b>0

Note: superscript * indicates the case in which retailers take CSR and ** indicates the case in which manufacturers take CSR; subscript m, r, and sc indicate that the decision makers are the manufacturer, the retailer, and the whole green supply chain, respectively; numbers 1, 2, and 3 indicate the game models under three different power structures: 1 represents the manufacturer-led decision scenario, 2 indicates the retailer-led decision scenario, and 3 is the Nash equilibrium decision scenario.

## Data Availability

Not applicable.

## Appendix A

Since the proof processes when the manufacturer dominates the market and when the retailer and the manufacturer jointly dominate the market are similar to that when the retailer dominates the market, and only the case when the retailer dominates the market is proved here. Separate partial derivatives of φ yield.

$$\frac{g_{2}^{*}}{\partial \varphi }=\frac{ab^{2}k_{1}z}{{\left[bz(4-\varphi )-(2k_{1}^{2}b+k_{2}^{2}z)\right]}^{2}}>0,\;\frac{w_{2}^{*}}{\partial \varphi }=\frac{ab^{2}z^{2}}{{\left[bz(4-\varphi )-(2k_{1}^{2}b+k_{2}^{2}z)\right]}^{2}}>0$$

$$\frac{v_{2}^{*}}{\partial \varphi }=\frac{abk_{2}z^{2}}{{\left[bz(4-\varphi )-(2k_{1}^{2}b+k_{2}^{2}z)\right]}^{2}}>0,\;\frac{\pi_{m_{2}}^{*}}{\partial \varphi }=\frac{a^{2}b^{3}z^{2}(2z-k_{1}^{2})}{{\left[bz(4-\varphi )-(2k_{1}^{2}b+k_{2}^{2}z)\right]}^{3}}>0,$$

$$\frac{\pi_{r_{2}}^{*}}{\partial \varphi }=\frac{2a^{2}b^{2}z^{2}}{{\left[2bz(4-\varphi )-2(2k_{1}^{2}b+k_{2}^{2}z)\right]}^{2}}>0,\;\frac{\pi_{SC_{2}}^{*}}{\partial \varphi }=\frac{a^{2}b^{2}z^{2}(8bz-4bk_{1}^{2}-bz\varphi -k_{2}^{2}z)}{2{\left[bz(4-\varphi )-(2k_{1}^{2}b+k_{2}^{2}z)\right]}^{3}}>0$$

$$\frac{p_{2}^{*}}{\partial \varphi }=\frac{-abz(bz-bk_{1}^{2}-k_{2}^{2}z)}{{\left[bz(4-\varphi )-(2k_{1}^{2}b+k_{2}^{2}z)\right]}^{2}}<0,\;\mathrm{End}\;\mathrm{of}\;\mathrm{proof}.\;$$

## Appendix B

Since the proof processes of retail price, green degree, and green marketing are similar to the proof process of wholesale price, only wholesale price is proved here.

The numerator of both is the same, both are $w_{2}^{*}$, $w_{3}^{*}$, and the denominator is $[bz(4-\varphi )-(2k_{1}^{2}b+k_{2}^{2}z)]-[bz(3-\varphi )-(k_{1}^{2}b+k_{2}^{2}z)]=b(z-k_{1}^{2})>0$, So $w_{r_{2}}^{*}<w_{N_{3}}^{*}$ is proven.

$$w_{1}^{*}-w_{2}^{*}=\frac{az[bz\varphi^{2}+(2bk_{1}^{2}+2bk_{2}^{2}z-4b^{2}z)\varphi +2bk_{1}^{2}k_{2}^{2}+k_{2}^{4}z-3b^{2}k_{1}^{2}-4bk_{2}^{2}z+4b^{2}z]}{\left[2bz(2-\varphi )-(k_{1}^{2}b+2k_{2}^{2}z)][bz(4-\varphi )-(2k_{1}^{2}b+k_{2}^{2}z)\right]},$$

let $bz\varphi^{2}+(2bk_{1}^{2}+2bk_{2}^{2}z-4b^{2}z)\varphi +2bk_{1}^{2}k_{2}^{2}+k_{2}^{4}z-3b^{2}k_{1}^{2}-4bk_{2}^{2}z+4b^{2}z=0$,

Since $k_{1}^{2}-z<0$, so the equation has no solution, $w_{1}^{*}>w_{2}^{*}$.

$w_{1}^{*}-w_{3}^{*}=\frac{az(b-b\varphi -k_{2}^{2})(2bz-bz\varphi -bk_{1}^{2}-k_{2}^{2}z)}{\left[2bz(2-\varphi )-(k_{1}^{2}b+2k_{2}^{2}z)][bz(3-\varphi )-(k_{1}^{2}b+k_{2}^{2}z)\right]}$, Because $\frac{k_{1}^{2}b+k_{2}^{2}z}{bz}<1$, therefore $2bz-bz\varphi -bk_{1}^{2}-k_{2}^{2}z>0$.

When $b-b\varphi -k_{2}^{2}>0$, $w_{1}^{*}\geq w_{3}^{*}>w_{2}^{*}$.When $\frac{b-k_{2}^{2}}{b}<\varphi <1$, $w_{3}^{*}>w_{1}^{*}>w_{2}^{*}$.

## Appendix C

Since the proof processes when the manufacturer dominates the market and when the retailer and the manufacturer jointly dominate the market are similar to that when the retailer dominates the market, only the case when the retailer dominates the market is proved here. The partial derivative of φ is obtained separately.

$$\frac{g_{2}^{**}}{\partial \varphi }=\frac{2ab^{2}k_{1}z}{{\left[2bz(2-\varphi )-(2k_{1}^{2}b+k_{2}^{2}z)\right]}^{2}}>0,\;\frac{v_{2}^{**}}{\partial \varphi }=\frac{2abk_{2}z^{2}}{{\left[2bz(2-\varphi )-(2k_{1}^{2}b+k_{2}^{2}z)\right]}^{2}}>0,$$

$$\frac{\pi_{m_{2}}^{**}}{\partial \varphi }=\frac{a^{2}b^{{}^{2}}z^{2}(4bz-2bz\varphi -2bk_{1}^{2}+k_{2}^{2}z)}{2{\left[2bz(2-\varphi )-(2k_{1}^{2}b+k_{2}^{2}z)\right]}^{3}}>0,\;\frac{\pi_{r_{2}}^{**}}{\partial \varphi }=\frac{4a^{2}b^{2}z^{2}}{{\left[4bz(2-\varphi )-2(2k_{1}^{2}b+k_{2}^{2}z)\right]}^{2}}>0,\;$$

$$\frac{\pi_{SC_{2}}^{**}}{\partial \varphi }=\frac{a^{2}b^{2}z^{2}(12bz-6bk_{1}^{2}-6bz\varphi -k_{2}^{2}z)}{2{\left[2bz(2-\varphi )-(2k_{1}^{2}b+k_{2}^{2}z)\right]}^{3}}>0,\;\frac{w_{2}^{**}}{\partial \varphi }=\frac{-abz(2bz-2bk_{1}^{2}-k_{2}^{2}z)}{{\left[2bz(2-\varphi )-(2k_{1}^{2}b+k_{2}^{2}z)\right]}^{2}}<0,\;$$

$$\frac{p_{2}^{**}}{\partial \varphi }=\frac{-2abz(bz-bk_{1}^{2}-k_{2}^{2}z)}{{\left[2bz(2-\varphi )-(2k_{1}^{2}b+k_{2}^{2}z)\right]}^{2}}<0,\;\mathrm{End}\;\mathrm{of}\;\mathrm{proof}.\;$$

## References

[1] Zhang Z., Yu L. (2021). Dynamic Optimization and Coordination of Cooperative Emission Reduction in a Dual-Channel Supply Chain Considering Reference Low-Carbon Effect and Low-Carbon Goodwill. Int. J. Environ. Res. Public Health.

[2] Woohyoung K., Kim H., Hwang J. (2020). Transnational Corporation’s Failure in China: Focus on Tesco. Sustainability.

[3] Marczewska M., Jaskanis A., Kostrzewski M. (2020). Knowledge, Competences and Competitive Advantage of the Green-Technology Companies in Poland. Sustainability.

[4] Küçükoğlu M.T., Pınar R.I. (2015). Positive Influences of Green Innovation on Company Performance. Procedia Soc. Behav. Sci..

[5] Zhang Z., Fu D., Zhou Q. (2020). Optimal Decisions of a Green Supply Chain under the Joint Action of Fairness Preference and Subsidy to the Manufacturer. Discret. Dyn. Nat. Soc..

[6] Chen S., Wang X., Ni L., Wu Y. (2017). Pricing Policies in Green Supply Chains with Vertical and Horizontal Competition. Sustainability.

[7] Zhu W., He Y. (2017). Green product design in supply chains under competition. Eur. J. Oper. Res..

[8] Sang S. (2017). Decentralized Channel Decisions of Green Supply Chain in a Fuzzy Decision Making Environment. Int. J. Comput. Intell. Syst..

[9] Yang D., Xiao T. (2017). Pricing and green level decisions of a green supply chain with governmental interventions under fuzzy uncertainties. J. Clean. Prod..

[10] Li Q., Xiao T., Qiu Y. (2018). Price and carbon emission reduction decisions and revenue-sharing contract considering fairness concerns. J. Clean. Prod..

[11] Swami S., Shah J. (2013). Channel coordination in green supply chain management. J. Oper. Res. Soc..

[12] Hong Z., Guo X. (2019). Green product supply chain contracts considering environmental responsibilities. Omega.

[13] Song H., Gao X. (2018). Green supply chain game model and analysis under revenue-sharing contract. J. Clean. Prod..

[14] Golden J.S., Subramanian V., Zimmerman J.B. (2011). Sustainability and commerce trends: Industry Consortia as the Drivers for Green Product Design. J. Ind. Ecol..

[15] Ni D., Li K. (2012). A game-theoretic analysis of social responsibility conduct in two-echelon supply chains. Int. J. Prod. Econ..

[16] Panda S. (2014). Coordination of a socially responsible supply chain using revenue sharing contract. Transp. Res. Part E Logist. Transp. Rev..

[17] Modak N.M., Panda S., Sana S.S. (2015). Pricing policy and coordination for a two-layer supply chain of duopolistic retailers and socially responsible manufacturer. Int. J. Logist. Res. App..

[18] Panda S., Modak N.M. (2016). Exploring the effects of social responsibility on coordination and profit division in a supply chain. J. Clean. Prod..

[19] Raza S.A. (2018). Supply chain coordination under a revenue-sharing contract with corporate social responsibility and partial demand information. Int. J. Prod. Econ..

[20] Song Z., Huang Y., Gu J. (2016). On Equilibrium Decisions of Socially Responsible Supply Chain with One Manufacture and Two Retailers. Chin. J. Manag..

[21] Wang Z., Wang M., Liu W. (2020). To introduce competition or not to introduce competition: An analysis of corporate social responsibility investment collaboration in a two-echelon supply chain. Transp. Res. Part E Logist. Transp. Rev..

[22] Li M., Xue J. (2016). Performance analysis of manufacturer collecting closed-loop supply chain under different channel power structures. Control Decis..

[23] Fan J., Liang X., Ni D. (2019). A Study on Corporate Social Responsibility and Product Quality in Supply Chains under Different Channel Power Structures. Chin. J. Manag..

[24] Ma P., Wang H., Shang J. (2013). Supply chain channel strategies with quality and marketing effort-dependent demand. Int. J. Prod. Econ..

[25] Gang X., Yue W., Wang S. (2014). Quality Improvement Policies in a supply chain with Stackelberg games. J. Appl. Math..

[26] Maiti T., Giri B.C. (2015). A closed loop supply chain under retail price and product quality dependent demand. J. Manuf. Syst..

[27] Ni D., Li K., Tang X. (2010). Social responsibility allocation in two-echelon supply chains: Insights from wholesale price contracts. Eur. J. Oper. Res..

[28] Yang Y., Chen Y., Chen S. (2019). Stimulating Corporate Social Responsibility in Supply Chain in Different Dominated Modes. Chin. J. Manag. Sci..

[29] Biswas I., Raj A., Samir K. (2018). Supply chain channel coordination with triple bottom line approach. Transp. Res. Part E Logist. Transp. Rev..

[30] Li B., Zhu M., Jiang Y., Li Z. (2015). Pricing policies of a competitive dual-channel green supply chain. J. Clean. Prod..

[31] Liu Y., Quan B.-T., Xu Q., Forrest J.Y.-L. (2019). Corporate social responsibility and decision analysis in a supply chain through government subsidy. J. Clean. Prod..

[32] Sang S., Zhang Q. (2020). Optimal Policies for Green Supply Chain with Corporate Social Responsible. J. Beijing Inst. Technol. (Soc. Sci. Ed.).

[33] Amaeshi K.M., Osuji O.K., Nnodim P. (2008). Corporate social responsibility in supply chains of global brands: A boundaryless responsibility? Clarifications, Exceptions and Implications. J. Bus Ethics.

[34] Ghosh D., Shah J. (2015). Supply chain analysis under green sensitive consumer demand and cost sharing contract. Int. J. Prod. Econ..

[35] Zhou Y., Bao M., Chen X., Xu X. (2017). Co-op advertising and emission reduction cost sharing contracts and coordination in low-carbon supply chain based on fairness concerns. Chin. J. Manag..

